# Synergy of single-cell sequencing analyses and *in vivo* lineage-tracing approaches: A new opportunity for stem cell biology

**DOI:** 10.32604/biocell.2022.018960

**Published:** 2022

**Authors:** Yuki MATSUSHITA, Wanida ONO, Noriaki ONO

**Affiliations:** University of Texas Health Science Center at Houston School of Dentistry, Houston, 77054, USA

**Keywords:** Single-cell sequencing, *in vivo* lineage-lineage, skeletal stem cells (SSCs), mesenchymal stem cells (MSCs), bone marrow stromal cells (BMSCs)

## Abstract

Single-cell sequencing technologies have rapidly progressed in recent years, and been applied to characterize stem cells in a number of organs. Somatic (postnatal) stem cells are generally identified using combinations of cell surface markers and transcription factors. However, it has been challenging to define micro-heterogeneity within “stem cell” populations, each of which stands at a different level of differentiation. As stem cells become defined at a single-cell level, their differentiation path becomes clearly defined. Here, this viewpoint discusses the potential synergy of single-cell sequencing analyses with *in vivo* lineage-tracing approaches, with an emphasis on practical considerations in stem cell biology.

## Introduction

Somatic stem cells play essential roles in tissue development and regeneration of postnatal animals. There are a variety of somatic stem cells in the body, such as in the blood, intestine, central nervous system, skin, hair follicle and bone (Gehart and Clevers, 2019; Gonzales and Fuchs, 2017; Matsushita *et al*., 2020b; Mendelson and Frenette, 2014; Metcalf, 2007; Mizuhashi *et al*., 2018; Méndez-Ferrer *et al*., 2010; Notta *et al*., 2016; Santos *et al*., 2018; Wei and Frenette, 2018). These cells are defined as cells capable of self-renewal, which is the ability to continue reproducing themselves, and multipotency, which is the ability to generate multiple types of differentiated cells (Blanpain *et al*., 2004). *In vivo* lineage-tracing approaches using a tamoxifen-inducible *creER-loxP* system, which is a reproducible method to track the whole life of a cell *in vivo*, have substantially contributed to the somatic stem cell research with the unparalleled capability to reveal stem cells’ dynamics within their native environment (Kretzschmar and Watt, 2012).

Here, we present our view on the utility of incorporating *in vivo* lineage-tracing approaches into single-cell sequencing omics studies. Single-cell sequencing analyses have rapidly progressed in the last five years, with incredible potential not only to unveil cellular diversity and distinct molecular signature of individual cells, but also to help place years of the previous research in an overall context within the tissue. Various approaches have been developed and become available to researchers, including single-cell RNA-seq, assay for transposase-accessible chromatin (ATAC)-seq, and their combined multiome seq (Liu *et al*., 2020; Stuart and Satija, 2019). These sequencing techniques have been applied to a diversity of research fields, including stem cell biology, developmental biology and cancer biology. One area that has lagged behind is the integration of a temporal factor, which typically relies on computational methods to infer dynamic lineage relationships within static samples. While some studies have attempted to resolve this issue by collecting samples at various time points, the differentiation pathway is largely inferred from computational predictions.

Here, we discuss the potential synergistic effect of *in vivo* lineage-tracing approaches and single-cell sequencing analyses in stem cell biology.

### Viewpoint

It is hypothesized that stem cells stand at the top of the hierarchy of a given cell lineage within a given tissue. A small number of stem cells are considered to provide the origin of many differentiated cell types downstream. In the field of skeletal stem cells, single-cell sequencing analyses have revealed the fundamental heterogeneity of bone marrow stromal cells; these analyses have computationally inferred the existence of stem cells within the lineage (Baryawno *et al*., 2019; Tikhonova *et al*., 2019; Wolock *et al*., 2019; Zhong *et al*., 2020). Practically, the key to success to interrogate stem cells by single-cell analyses is how efficiently the target stem cell populations can be enriched prior to loading on to the single-cell sequencing platforms. The bone marrow is predominantly composed of non-skeletal/mesenchymal cells including blood cells and vascular cells; therefore, bone marrow stromal cells and their subset skeletal stem cells represent only an extremely small fraction −0.001~0.01% – of all bone marrow cells (Pittenger *et al*., 1999). In order to selectively harvest this small amount of “stem cell populations”, target cells need to be isolated by a panel of cell surface markers or fluorescent proteins such as GFP for fluorescence-activated cell sorting (FACS) (Chan *et al*., 2015; Chan *et al*., 2018; Matsushita *et al*., 2021c; Morikawa *et al*., 2009). This step can be used to remove the “contaminants” – hematopoietic and vascular cells – so that we can sufficiently enrich stem cells for downstream analyses (Matsushita *et al*., 2021a).

There are two distinct ways to remove contaminant cells and enrich putative stem cell populations. A positive selection method uses a combination of markers that are expressed by target cell populations to capture stem cells, while a negative selection method uses a combination of markers that are not expressed by target cell populations to exclude contaminant cells. For example, mouse SSCs are negatively selected at first as CD45^−^CD31^−^Ter119^−^, and then purified using both positive and negative markers, as CD51^+^CD90^−^CD105^−^CD200^+^ (Chan *et al*., 2015). There are certain advantages and disadvantages of positive and negative selection. Positive selection with “stem cell markers” allows to capture only a small and limited group of cells for downstream analyses. It is therefore possible that other important stem cell populations can be excluded from the analyses. In contrast, negative selection aiming to remove contaminant cells allows to capture a wide breadth of relevant cell types. However, it is possible that the important stem cell populations become underrepresented due to an exceeding number of non-stem cell populations. Although cell sorting with negative selection is generally associated with a greater level of contamination of wanted cell types, cell sorting with positive selection also inevitably involves with at least to some degrees of contamination. It is therefore important to combine these two selection methods to enrich the intended cell population.

The enriched populations of “stem cells” can be subjected to single-cell sequencing analyses, although such analyses will almost always discover substantial heterogeneity within the given populations. The putative trajectories of stem cell differentiation can be inferred from single-cell sequencing data; however, the inherent limitation of this approach is that cell surface markers or fluorescent proteins used to enrich these cells can only capture a snapshot of the cell population involved in the dynamic process of tissue remodeling.

How can we add a temporal factor to this analysis to account for the dynamic change of the cell populations over time? *In vivo* lineage-tracing approach using a tamoxifen-inducible *creER-loxP* system with a fluorescent reporter such as a *Rosa26-loxp-stop-loxp-tdTomato* allele (Madisen *et al*., 2010) can be used to track the fate of a cell through the entire life. The fluorescent protein is permanently expressed by the cell that has undergone recombination, which can be temporarily induced in the presence of tamoxifen specifically within the cells in which creER proteins are present. Because the recombination is irreversible and occurs in the genome, the targeted cells undergoing the removal of the stop cassette continue to express the fluorescent protein even after subsequent replication and differentiation of daughter cells. This enables selective marking of cells expressing the “driver” gene, as well as their descendant cells, which differentiated from the originally targeted cells.

To successfully define the differentiation pathway of stem cells using *in vivo* lineage-tracing approaches, it is extremely important to select the right *creER* line with the right “driver” gene. Ideally, the “driver” gene should target only stem cells, but not their downstream populations. This would allow to visualize the *in vivo* lineage progression of stem cells over time. For example, parathyroid hormone-related protein (PTHrP) is an ideal marker for skeletal stem cells in the growth plate. *Pthrp-creER* line specifically marks slow-cycling chondrocytes in the resting zone of the growth plate (Mizuhashi *et al*., 2018), and only a small subset of these cells undergo asymmetric cell divisions and differentiate into columnar chondrocytes, making it an ideal model to track the fate of somatic stem cells at single-cell levels. In contrast, the Rosa26 locus is ubiquitously active. When *Rosa26* is used as the driver (Ventura *et al*., 2007), essentially all the cells, including stem cells and their descendants, are simultaneously labelled, making it impossible to define the precursor-product relationship. These two extreme scenarios give us the examples how important it is to select the right “driver” gene for *in vivo* lineage-tracing experiments.

We need to keep in mind that, despite the substantial utility, *in vivo* lineage-tracing approaches have several caveats. First, due to inherent ineffectiveness, the tamoxifen-inducible *creER-loxP* system induces recombination only in a small subset of target cell populations. It is important to verify the recombination efficiency in a target cell population, and its correlation with endogenous gene expression using immunohistochemistry or more reliable knock-in fluorescent reporter lines on the short-chase samples. Second, it is possible that tamoxifen-induced recombination can artificially select a certain stem cell population. Third, tamoxifen may have its own adverse effects on a variety of tissues, such as brains (Lee *et al*., 2020).

In contrast, constitutively active *cre* lines that do not require tamoxifen injection to label stem cells and their descendants. Constitutively active *cre* lines with “driver” genes active in putative stem cell populations, such as *Prrx1-cre* (Logan *et al*., 2002), *Lepr-cre* (Zhou *et al*., 2014) and *Ctsk-cre* (Debnath *et al*., 2018) in the skeletal stem cell field, have been utilized to define the stem cell function in a variety of contexts. However, the inherent limitation of this system is that there is no “temporal” control in recombination, making it impossible to determine when differentiation from stem cells to differentiated cells have occurred. Therefore, it requires extreme caution to make conclusions on the lineage relationship among diverse groups of cells.

By applying single-cell sequencing analyses to lineage-marked cells, particularly those marked by the tamoxifen-inducible *creER-loxP* system, we can add a temporal factor and capture the snapshots of cells at various stages of differentiation at a single cell level (Kester and van Oudenaarden, 2018; Wagner and Klein, 2020). These combinatory methods are useful in unraveling dynamic cellular events, such as stem cell differentiation in normal tissue growth and regeneration.

**FIGURE 1.**
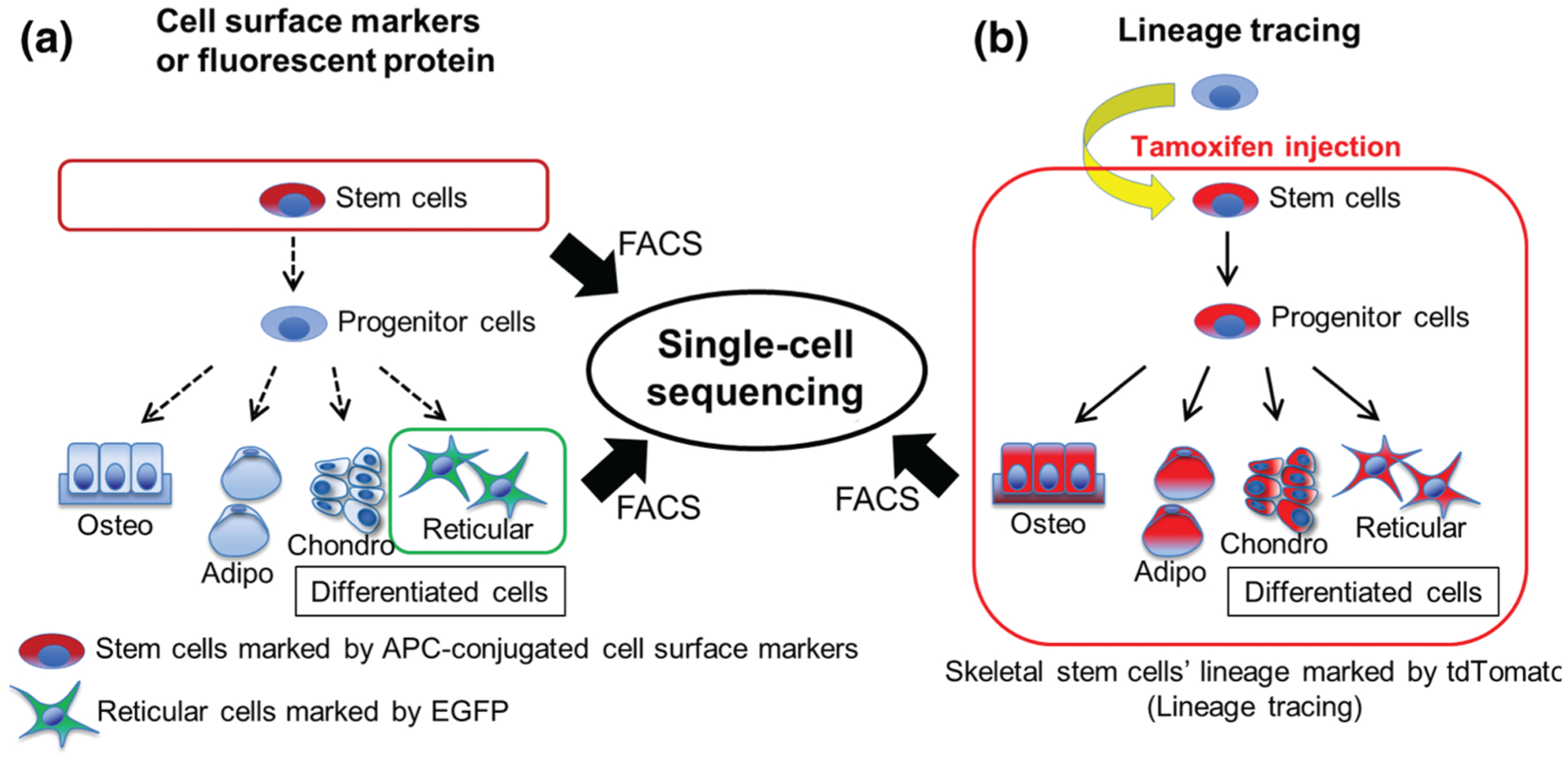
Single-cell sequencing analysis of the target cells enriched by fluorescence-activated cell sorting (FACS). (a) Cell surface markers or fluorescent reporter proteins can mark a group of cells expressing the marker genes at a given time (“*Snapshot*”). (b) *In vivo* lineage-tracing system can mark a specific group of cells descended from a specific type of cell, therefore including a “*temporal factor*”. Single-cell sequencing analyses can reveal the heterogeneity of the lineage-marked cells with inference to precursor-product relationships.

**FIGURE 2.**
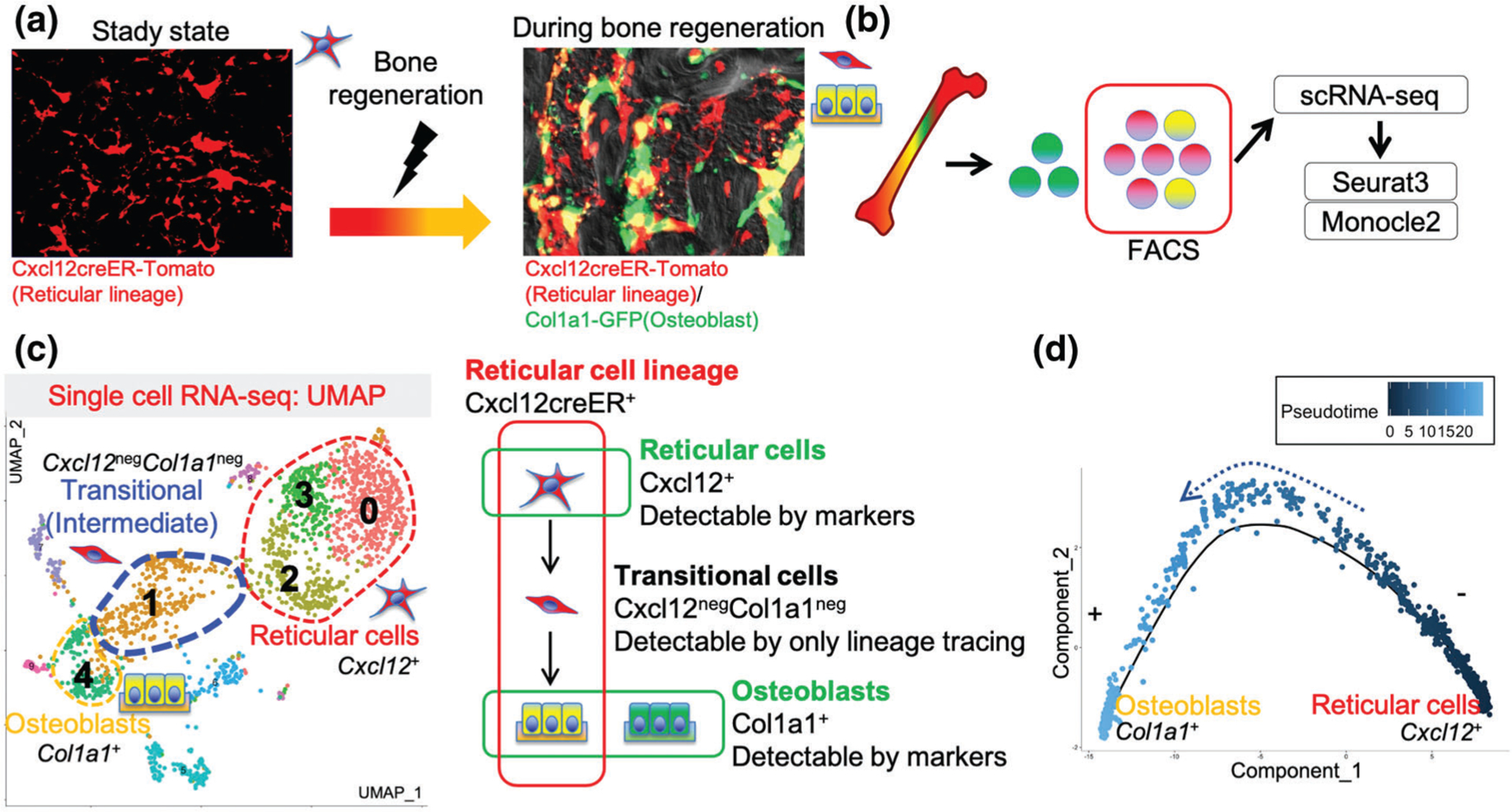
Actual strategy for the combination of lineage tracing and single-cell sequencing analysis using bone regeneration model. (a) Cxcll2^+^ reticular cells’ descendants (red) contribute to osteoblasts in regenerating bone (yellow). Osteoblasts marked by green are from other cell origins. (b) Sorting only Cxcll2^+^ lineage cells (red and yellow) using FACS and loading to single-cell sequencing platforms. (c) Left: UMAP based visualization. Cxcll2^+^ reticular cells differentiate into osteoblasts through the undefined transitional cells first revealed by single-cell analyses. Right: Target cell populations marked by marker genes or lineage tracing. (d) Pseudo-time analyses show the trajectory from Cxcll2^+^ reticular cells to Colla1^+^ osteoblasts. Single-cell RNA-seq plots reproduced and adapted with permission from Matsushita *et al. Nature communications*, 2020 (CC BY 4.0).

Generally speaking, cell surface markers or fluorescent reporter proteins are expressed by a given specific cell type at the time of analysis. In contrast, *in vivo* lineage-tracing approaches can mark the entire lineage including stem cells and all of their descendant cells, at least theoretically (Fig. 1). By taking advantage of a combinatory lineage-tracing and single-cell RNA-seq approaches, we recently demonstrated a new concept in bone regeneration (Matsushita *et al*., 2020a) using a *Cxcl12-creER* transgenic line that is specifically active in quiescent pre-adipocyte-like marrow stromal cells. This combinatory approach allowed us to identify a previously uncharacterized “intermediate” cell populations (Fig. 2). Although a small number of skeletal stem cells had been thought to be solely responsible for bone regeneration, our new findings shed light on the possibility that their downstream terminally differentiated cells are also involved in bone regeneration through the mechanism involving cellular plasticity (Matsushita *et al*., 2021b).

Pseudo-time analyses predict the differentiation trajectory of the single cells based on the single-cell sequencing data, in which a longer trajectory is indicative of a more difficult differentiation pathway. However, pseudo-time analyses sometimes may conflict with the actual known lineage dynamics. It might be possible in the future through innovative computational methods to determine how the actual rate of cell differentiation observed by lineage-tracing differs from pseudo-time-based computational predictions, and facilitate the development of more accurate computational modalities. Moreover, single-cell seq analyses can be performed in multiple time points before and after the event, and then multiple single-cell seq data can be computationally combined. The trajectory of cell differentiation can be estimated by Pseudo-time analysis in a “static” single-cell sequencing dataset; however, the relevance of the results can be greatly improved by combining these with “real-time” data from biological samples from several time points. In the referenced case, “real-time” single-cell seq analyses in multiple sequential time points lead to the assumption that the intermediate state cells that “de-differentiated” from Cxcl12^+^ reticular cells can “re-differentiate” into Col1a1^+^ osteoblasts (Fig. 2). The combination of lineage-tracing and single-cell sequencing analyses has been used in various fields, including in neuron, bone marrow, heart and brain (Figueres-Oñate *et al*., 2021; Magnusson *et al*., 2020; Su *et al*., 2018; Tikhonova *et al*., 2019). Although the existing computational trajectory analysis tools could resolve lineage differentiation at least to some extent in these studies, there still remains substantial room for improvement in order to accurately predict *in vivo* lineage progression of individual cells over multiple time points.

The advantage of the *in vivo* lineage-tracing approach based on the tamoxifen-inducible *creER-loxP* system is its versatility. The differentiation stage of lineage-marked cells can be further stratified by standard immunofluorescent staining or other cell type-specific transgenic marker genes. In other instances, the Rosa26 reporter gene for *creER-loxP* recombination can be altered to a multicolor format such as to the *Rosa26-Confetti* (Livet *et al*., 2007) allele that permits *in vivo* clonal analysis. These additional modalities could be further combined with single-cell sequencing approaches to facilitate the understanding of stem cell behaviors *in vivo*.

“Drop-out”, in which a gene is observed at a moderate or high expression level in one cell but is not detected in another cell, is the inherent weakness of single-cell sequencing analyses, especially those utilizing drop-seq based approaches (Kharchenko *et al*., 2014). It occurs due to the low abundance of mRNAs in individual cells and the inefficient capture of the transcriptome, as well as the stochasticity of mRNA expression (Qiu, 2020). Importantly, the drop-out rate is potentially higher on “stem cells” because of low mRNA levels and small numbers of expressed genes, compared to differentiated cells (Islam *et al*., 2011). It remains to be determined whether *in vivo* lineage-tracing approaches can help to resolve this issue by knowing which progenitor cell subpopulation preferentially differentiates down different lineages.

### Vision of the Future

A variety of methods for single-cell sequencing analyses have been developed in recent years, making it possible to infer cellular dynamics in a more detailed manner. Multiome analyses, including those measuring both RNA and chromatin accessibility simultaneously, facilitate more detailed characterization of individual cells. A number of innovative algorithms facilitating single-cell computational analyses have been developed (Liu *et al*., 2020; Stuart and Satija, 2019). With a myriad of single-cell computational approaches available, what is needed in the future is a strategy for high-dimensional integrative analyses. Because single-cell sequencing analysis alone can only provide an estimated landscape of diverse cell populations, validation is the key step to sustain the authenticity of the computational findings. Using identified cell type-specific markers, we can analyze specific cell populations. Subsequently in combination with *in vivo* lineage-tracing approaches, we can dissect cellular dynamics of a given cell population in time and space. This combinatory approach can be applied to multiple scenarios, to understand fundamental biological phenomena or evaluate therapeutic responses. Furthermore, the combination of single-cell sequencing and *in vivo* cell lineage approaches can facilitate deeper understanding of native stem cells, not only in the context of tissue growth and homeostasis, but also in the context of more dynamic situations involving stem cell mobilization, such as tissue regeneration, tumor growth and drug response; these interventions are likely to induce drastic changes to somatic stem cells. Single-cell sequencing analyses can also potentially unravel multicellular dynamics. By applying *in vivo* lineage-tracing approaches to disease models, the impact of genetic diseases on cell lineages can be investigated. We expect that the in-depth biological information obtained from *in vivo* cell-lineage analyses will synergize well with single-cell sequencing analyses and improve their accuracy.

Currently, more refined techniques for *in vivo* lineage-tracing approaches have been developed, including those using the barcode technology in which the clonal information is encoded by DNA sequence barcodes. This barcode-based cell marking can theoretically allow unlimited numbers of clonal labeling, unlike fluorescence-based cell marking with a limited number. It is anticipated that barcode-based lineage-tracing approaches using the CRISPR/Cas9 system will become an important modality in the field in stem cell biology (Wagner and Klein, 2020).

Taken together, we conclude that the combination of single-cell analyses and *in vivo* lineage-tracing approaches produce a substantial synergistic effect in advancing stem cell biology.
